# Alterations in candidate genes PHF2, FANCC, PTCH1 and XPA at chromosomal 9q22.3 region: Pathological significance in early- and late-onset breast carcinoma

**DOI:** 10.1186/1476-4598-7-84

**Published:** 2008-11-06

**Authors:** Satyabrata Sinha, Ratnesh K Singh, Neyaz Alam, Anup Roy, Susanta Roychoudhury, Chinmay Kumar Panda

**Affiliations:** 1 Department of Oncogene Regulation, Chittaranjan National Cancer Institute, Kolkata, India; 2 Department of Surgical Oncology, Chittaranjan National Cancer Institute, Kolkata, India; 3 Department of Pathology, Medical College, Kolkata, India; 4 Molecular and Human Genetics Division, Indian Institute of Chemical Biology, Kolkata, India

## Abstract

**Introduction:**

Younger women with breast carcinoma (BC) exhibits more aggressive pathologic features compared to older women; young age could be an independent predictor of adverse prognosis. To find any existing differences in the molecular pathogenesis of BC in both younger and older women, alterations at chromosomal (chr.) 9q22.32-22.33 region were studied owing to its association in wide variety of tumors. Present work focuses on comparative analysis of alterations of four candidate genes; PHF2, FANCC, PTCH1 and XPA located within 4.4 Mb region of the afore-said locus in two age groups of BC, as well as the interrelation and prognostic significance of alterations of these genes.

**Methods:**

Deletion analysis of PHF2, FANCC, PTCH1 and XPA were examined in a subset of 47 early-onset (group-A: ≤ 40 years) and 59 late-onset (group-B: > 40 years) breast carcinomas using both microsatellite and exonic markers. Methylation Sensitive Restriction analysis (MSRA) was done to check for promoter methylation. Quantitative real-time polymerase chain reaction (Q-PCR) and immunohistochemisty (IHC) was done in some genes to see their relative mRNA and protein expressions respectively. Clinico-pathological correlation of different parameters as well as patient survival was calculated using different statistical softwares like EpiInfo 6.04b, SPSS 10.0 etc.

**Results:**

Either age group exhibited high frequency of overall alterations in PHF2, FANCC and PTCH1 compared to XPA. Samples with alteration (deletion/methylation) in these genes showed reduced level of mRNA expression as seen by Q-PCR. Immunohistochemical analysis of FANCC and PTCH1 also supported this observation. Poor patient survival was noted in both age groups having alterations in FANCC. Similar result was also seen with PTCH1 and XPA alterations in group-A and PHF2 alterations in group-B. This reflected their roles as prognostic tools in the respective groups in which they were altered.

**Conclusion:**

Overall alterations of PHF2, FANCC and PTCH1 were comparatively higher than XPA. Differential association of alterations in FANCC and PTCH1 with that of PHF2, XPA and two breast cancer susceptibility genes (BRCA1/BRCA2) in the two age groups suggests differences in their molecular pathogenesis and dysregulation of multiple DNA repair pathways as well as hedgehog dependent stem cell renewal pathway.

## Introduction

Breast carcinoma (BC) is the second leading cause of cancer deaths and the most common cancer among women. About 23% of urban women in eastern India are affected by this disease [1]. Depending on age at onset, BC can be early-onset (≤ 40 years) and late-onset (> 40 years) type. However, the cut-off value for early-onset BC varies among investigators, ranging from 35–50 years. Significant differences in clinico-pathological features like large tumor size of higher grade, presence of positive lymph nodes, absence of steroid receptors, and high S-phase fraction in younger women with BC indicated altered biology and pathogenesis between these two groups of BC [2-4]. Another report showed that in Asian population, BC patients below 40 years have tumors with a poorer prognostic profile. However, this did not translate into a poorer overall survival, and this might be attributable to more aggressive adjuvant treatment of younger patients [5]. All these reports suggested early-onset BC to be a biologically separate disease and independently predict more adverse outcomes. Thus, the molecular analysis of BC in the two age groups is pertinent to understand the differences in pathogenesis, if any. Our earlier studies on chromosomes (chrs.) 1p/q, 9p and 11p/q showed differential pattern of molecular alterations between the two age groups of BC [3-6]. In order to understand the pathogenesis in detail our aim is to analyze the alterations in other chromosomal regions which showed frequent alterations in BC.

Cytogenetic analyses have revealed a variety of chromosomal aberrations at chr.9q22 in different malignancies including BC [7]. Comparative genomic hybridization (CGH) and flow-cytometric analyses also showed losses at chr.9q in BC [8-10]. Studies in basal cell carcinoma (BCC), squamous cell carcinoma (SCC) as well as bladder, prostate, esophageal and blood cancer detected frequent loss of heterozygosity (LOH) at chr.9q22.3 [11,12]. Significant correlation between losses of chr.9q22.3 with lymph node metastasis in BC is reported [13].

Chr.9q22.3 is a relatively broad region (8.71 Mb) harboring several putative tumor suppressor genes (TSGs). We focused primarily on a 4.4 Mb region between the chr.9q22.32-22.33 where DNA-damage repair genes like FANCC (Fanconi anaemia Complementation-group C), XPA (Xeroderma Pigmentosum A) as well as the hedgehog (HH) pathway associated gene PTCH1 (*human homologue of Drosophila patched gene*) are localized [13-16]. FANCC localized at chr.9q22.32 (96.89 Mb from p-ter) is associated with reconstitutive and self-renewal potential of haematopoietic stem cells [17,18]. Its ablation leads to increased sensitivity to DNA crosslinking agents, loss of FANCD2 activation, and impaired homologous recombination due to poor functioning of the BRCA1-BRCA2-NBS1-RAD51 mediated DNA repair [19]. Deletion of FANCC in bladder carcinoma, mutations in young-onset pancreatic cancer and Fanconi Anaemia-C patients, and down-regulation in head and neck squamous cell carcinoma (HNSCC) are reported [16,20-22]. PTCH1 (350 Kb downstream to FANCC) is a key regulator in stem cell renewal hedgehog signaling pathway and functions as a tumor suppressor by inhibiting G_1_-S and G_2_-M phases of cell cycle. PTCH1 has been implicated in the development of medulloblastomas, glioblastomas, rhabdomyosarcomas, pancreatic cancers, and other soft tissue tumors [23]. A recent report suggested loss of PTCH1 in 19% of human breast cancers, and 33% of breast cancer cell lines [9]. Mutations in PTCH1 gene have been reported in nevoid basal cell carcinoma syndrome (NBCCS), familial BC and its loss induced tumor progression in basal cell carcinoma (BCC) [24,25]. Furthermore, immunohistochemical analysis showed reduced expression of PTCH1 due to promoter methylation in BC [26]. XPA (2.23 Mb down-stream of PTCH1) gene is essential for assembly of the pre-incision complex during the processing of DNA damage via the nucleotide excision repair (NER) pathway [27-29]. Defects in NER are associated with several human autosomal recessive hereditary disorders, such as xeroderma pigmentosum and a marked predisposition to skin cancer (early BCC, SCC and melanoma); early internal tumors [30]. LOH in sporadic ovarian, colon, lung carcinomas and significant association between oral premalignant lesions and polymorphism of XPA are reported [31,32]. PHF2, a novel transcriptional regulatory gene associated with hereditary sensory neuropathy type 1 (HSN1) has also been identified at chr.9q22.32, 1.52 Mb upstream of FANCC [33]. However, its role in tumorigenesis is not yet well understood.

Thus, it is evident that alterations of these genes together could cumulatively increase genetic instability and cellular proliferation. Furthermore, study of co-alterations of these genes could be useful in diagnosis and prognosis of a disease. Thus an attempt was made in this study to analyze the alterations (deletion, methylation and expression) of PHF2, FANCC, PTCH1 and XPA in early- and late-onset BC of Indian patients and correlate them with clinico-pathological differences.

## Methods

### Patients, Tumor tissues and cell lines

One hundred six invasive breast carcinomas (BC) and their corresponding normal tissues or peripheral blood leukocytes (PBL) were randomly collected from 104 unrelated patients, undergoing surgery at the hospital of Chittaranjan National Cancer Institute, Kolkata, India and stored at -80°C over a period of 9 years (1999–2007). The BC samples were segregated into early-onset BC (group-A: ≤ 40 years) and late-onset BC (group-B: > 40 years) depending on the age of the patient at onset of tumor. Informed consent from patients and Research Ethics Committee of the institute were obtained. All these tumors were graded and staged according to UICC TNM classification [34]. Detailed clinico-pathological histories of the patients are presented in Table 1. Part of the freshly operated tissues was directly collected in TRIzol reagent (Invitrogen, USA) for RNA isolation and the rest for DNA extraction. The BC cell line MCF-7 was obtained from American Type Culture Collection and grown according to ATCC instructions.

**Table 1:** Clinico-pathological parameters of early- and late-onset BC

	Group A			Group B		
		
Clinical features	Case	Mean age (years)	Age range (years)	Case	Mean age (years)	Age range (years)
Histological Type						
Ductal	45	34	25–40	59	53	41–77
Lobular	2	28	20–35	0	_	_
						
Clinical Stage						
TNM Stage I	1	40	40	2	48	45–50
TNM Stage II	7	33	23–40	21	51	42–61
TNM Stage III	36	35	20–40	33	53	41–77
TNM Stage IV	3	37	36–38	3	57	48–72
						
Tumor Differentiation						
Grade 1	4	32	20–40	8	52	43–61
Grade 2	32	33	18–40	39	47	41–42
Grade 3	11	38	33–40	12	56	42–77
						
Lymph Node						
Positive	33	35	26–40	48	52	42–77
Negative	14	31	18–40	11	51	41–65

### Microdissection and DNA Extraction

Contaminant normal cells in the samples were removed by manual microdissection from cryosections (5 μm) using surgical knives under a dissecting microscope [Leica MZ 16]. The representative sections from different regions of the specimens were stained with hematoxylin-eosin for diagnosis as well as for marking of tumor-rich regions. The microdissected samples containing at least 70–80% tumor cells were taken for DNA isolation via proteinase-K digestion, followed by phenol/chloroform extraction procedure [35].

### Deletion analysis of the candidate TSGs

Deletion analysis of the candidate genes was done using 6 microsatellite markers in the following order: Centromere- (PHF2: D9S1803, D9S197, D9S196), (FANCC: D9S1958), (PTCH1: D9S1816) and (XPA: D9S1714) – Telomere, selected on the basis of their map positions and heterozygosity (Ensembl release 44; Genome Database). One exonic marker each from PHF2 exon-18 and FANCC exon-2 were designed using Primer3Input 0.4.0 software to see the homozygous/hemizygous deletion (HD/HED) status of these genes [Table 2].

**Table 2:** Summary of oligonucleotides

**Primers**	**Location**	**Analytical purpose**	**Forward Primer**	**Reverse Primer**	**Size (bp)**
PHF2 Ex-18	9q22.32	HD/HED	5'-ACTCCTGCCTGCAGACCAC-3'	5'-CCTGCTCTTCCTCGTAGTCG-3'	168
FANCC Ex-2	9q22.32	HD/HED	5'-TGGCTCAAGATTCAGTAGATCTTTC-3'	5'-TTTCAAGGCTTCATACATCTTCC-3'	158
PHF2 promoter	-893 to -670 region	Methylation	5'-GTGGTGTCCCAACCAGAAAC-3'	5'-GGGACCCCGAGGATAAGATA-3'	223
FANCC promoter	-319 to -144 region	Methylation	5'-TTTTACCCCGTTGACAAAGC-3'	5'-CGGTACTGCTCCAGTGTTCC-3'	175
PTCH1 promoter	-715 to -420 region	Methylation	5'-CGAGGAGCACAAGAAAGCAG-3'	5'-AGAAAGAGCCAGCGAATCC-3'	295
XPA promoter Ex.1	-126 to +56 region	Methylation	5'-AGGCGCTCTCACTCAGAAAG-3'	5'-TCCGCGGGTTGCTCTAAA-3'	182
PTCH1 Ex.*23	Intron 22–23 to Ex.23	SSCP	5'-TCTAACCCACCCTCACCCTC-3'	5'-ATTGCTAGGGCCAGAATGCC-3'	226
PTCH1 Ex.23	Ex.23 to Intron 23–24	SSCP	5'-TTCTGCCTCCGTGACTGTC-3'	5'-CTCTAGGTCCCTTGGCTGC-3'	265
PHF2 Ex. 18–19	Coding regions of exons	Q-PCR	5'-ACTCCTGCCTGCAGACCAC-3'	5'-TCGACCGGGACTTAAAGATG-3'	257
FANCC Ex. 7–8	Coding regions of exons	Q-PCR	5'-TGGAGGCTCTCCTCATCTGT-3'	5'-GCATTCGATCCTTCTCAGACA-3'	223
PTCH1 Ex. 1–2	Coding regions of exons	Q-PCR	5'-GACCGGGACTATCTGCA-3'	5'-GAGGAGGCCCACAACC-3'	186
β2 Microglobulin	15q21-q22.2	Q-PCR	5'-GTGCTCGCGCTACTCTCTCT-3'	5'-TCAATGTCGGATGGATGAAA-3'	153
β-3A-adaptin (K1*)	5q14.1	DNA cleavage control	5'-TGCCCTCTGGACTGGAACCT-3'	5'-CCTGAGCCCAGCCCAAGTC-3'	445
RARβ2 (K2**)	3p24.2	DNA integrity control	5'-AGAGTTTGATGGAGTTGGGT-3'	5'-CATTCGGTTTGGGTCAATCC-3'	229

To detect for deletion in informative microsatellite markers, standard polymerase chain reaction (PCR) analysis with [γ-p32] ATP end labeled forward primer was done in a 20 μl reaction volume as described previously [35,36]. Multiplex PCR was done to see deletion (HD/HED) in the non-informative markers [35]. The D9S104 marker at chr.9p13 was used as control (CL) due to low frequency of alterations seen in BC (data not shown). The PCR products were run on 2% agarose gel, stained with ethidium bromide and visualized under UV transluminator.

Using a densitometric scanner (Bio-Rad GS-800, USA) the number of alleles at LOI (locus of interest) was determined from the signal intensities according to the following formula:

$$\text{Allele Number}=2\times \frac{\text{LOI}(\text{T})/\text{CL}(\text{T})}{\text{LOI}(\text{N})/\text{CL}(\text{N})}$$

Where T = tumor DNA; N = normal DNA; CL = Control; LOI = Locus of Interest

Allelic values of ≤ 0.5, 0.9 – 1.7, ≥ 1.8 to < 5.0 and > 5.0 will be considered as homozygous deletion (HD), hemizygous deletion (HED), retention of homozygosity (RA) and amplification of the alleles respectively [37].

### PCR based Methylation sensitive restriction analysis (MSRA)

Promoter methylation of PHF2, FANCC, PTCH1 and XPA were done in all BC samples by MSRA using methyl-sensitive restriction enzyme HpaII and its methylation in-sensitive iso-schizomer MspI [38]. Primer sequences are listed in Table 2. Promoter region of PTCH1 gene harbored 6 CCGG-recognition sites for the above restriction enzymes while, there were two CCGG sites in FANCC promoter and one each in PHF2 and XPA gene promoters. The 445-bp fragment of β-3A adaptin gene (K1) and 229-bp fragment of RARβ2 exon-1 (K2) was used as digestion and integrity controls respectively [39].

### Mutation analysis

Mutation analysis of exon-23 of PTCH1 was done in 60 BC samples by single strand conformation polymorphism (SSCP) analysis using [α-p32] dCTP as described previously [36]. Two primers from exon 23 (Ex*23 and Ex-23) were designed to cover the entire exon-23 which is a mutational hotspot and reported to be mutated in a variety of cancers [Table 2]. Sequencing for both strands of the samples showing abnormal band shift in autoradiographs were done using 3100-Avant Genetic Analyzer (PE Applied Biosystems Inc, USA).

### Quantitative Real-time PCR (Q-PCR)

The mRNA expression of PHF2, FANCC, PTCH1 and XPA in normal breast tissue (n = 6), and primary BC (n = 14) and 1 cell line (MCF-7) was analyzed by quantitative real-time (Q-PCR). Total RNA was isolated from samples using TRIzol reagent according to the manufacturer's protocol (Ambion Inc.). Complementary-DNA (cDNA) was synthesized from 1 μg total RNA using Random hexamer (Invitrogen, USA) and Superscript III (Invitrogen, USA) according to manufacturer's protocol. PCR was performed in 40 cycles on an ABI Prism 7500 using Power SYBR Green PCR Master Mix (Applied Biosystems, USA) in a final volume of 15 μl. Each sample was run in triplicates and average value was considered for analysis. Relative quantities (ΔΔCt) were obtained by normalization against human beta2-microglobulin gene [40]. Primers are listed in Table 2.

### Immunohistochemistry

Immunostaining of FANCC and PTCH1 protein were done in 20 primary breast carcinomas using the Avidin-Biotin-Peroxidase Complex (ABC) staining kit according to manufacturers protocol (Santa Cruz Biotechnology, CA, USA). About 3–5 μm paraffin sections were de-paraffinized, rehydrated and reacted overnight with primary antibodies (goat polyclonal IgG, sc-18110 for FANCC (C-14) and sc-6149 for PTCH1 (G-19); Santa Cruz, CA, USA) at a dilution of 1:100 at 4°C. Horse-Radish-Peroxidase conjugated rabbit anti-goat secondary antibody (sc-2768) was added at 1:500 dilutions. The slides were developed using 3-3' diaminobenzidine (DAB) as the chromogen and counterstained with hematoxylin. The staining intensity (- = negative, + = weak, 2+ = moderate, and 3+ = strong) and the percentage of positive cells (- = 0%, + = 1–24%, 2+ = 25–49%, and 3+ = 50–100%) were evaluated by two observers independently [41].

### Statistical Analysis of Clinical Data

Fisher's exact test was used to determine the association between tumors genetic profile and different clinico-pathological features. Survival analysis was performed according to Kaplan-Meier method. Post-operative overall survival was measured from the date of surgery to the date of last follow-up or death (up to 5 years). Probability value (P-value) ≤ 0.05 was considered statistically significant. All the statistical analysis was performed using statistical programs EpiInfo 6.04b, SPSS 10.0 (SPSS Inc. Chicago, IL, USA).

## Results

### Deletion analysis of candidate TSGs loci

**Figure 1:**
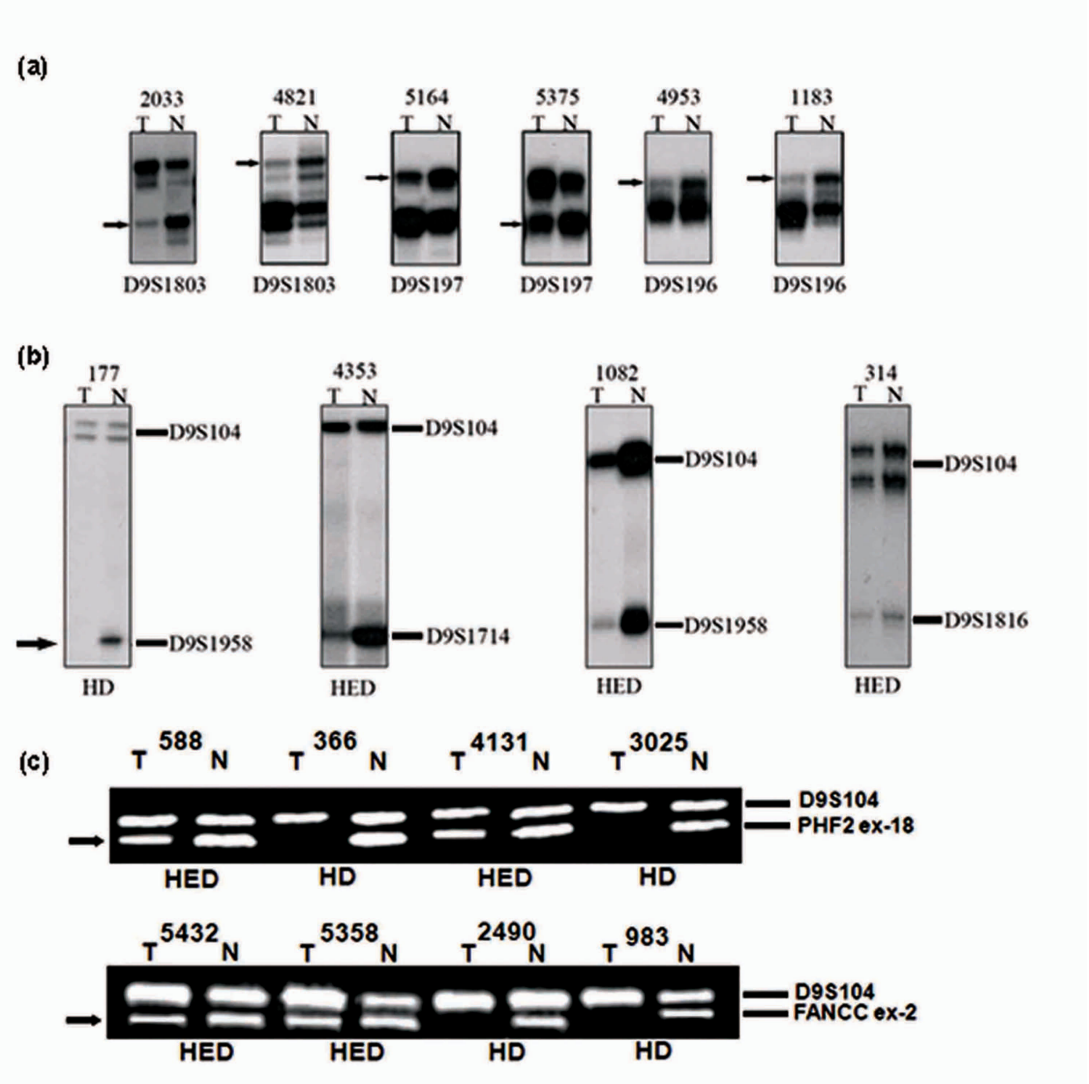
**(a) Representative autoradiograph showing loss of heterozygosity (LOH) (b) homozygous/hemizygous deletion (HD/HED) analysis in microsatellite markers (c) HD/HED of exonic markers.** → indicates loss of corresponding alleles.

**Figure 2:**
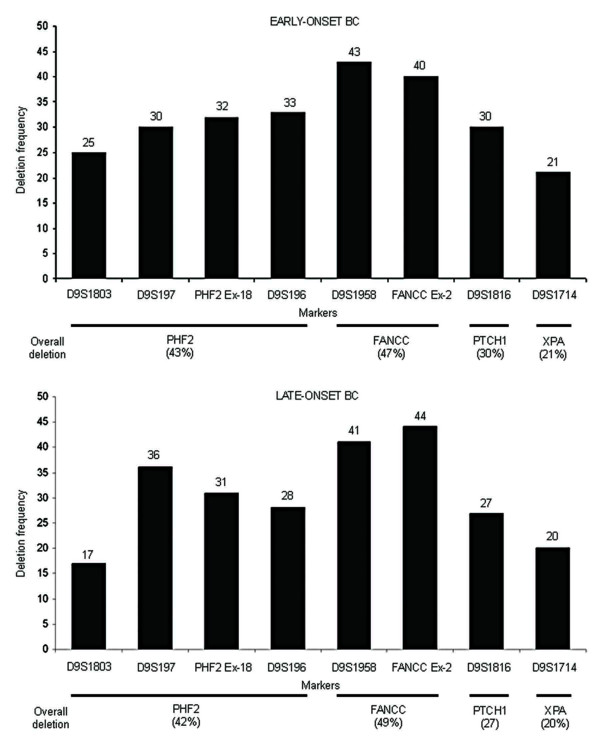
Pattern of deletion at different chr.9q22.32-22.33 markers in early- and late-onset BC.

Deletion mapping identified LOH in microsatellite markers and both hemi/homozygous deletion in the exonic markers [Figure 1]. Compared to XPA, high deletion was seen in the PHF2, FANCC and PTCH1 loci in both age groups of BC [Figure 2; Additional Files 1 and 2]. Order of deletion frequencies were: group-A: FANCC (47%) > PHF2 (43%) > PTCH1 (30%) > XPA (21%) and group-B: FANCC (49%) > PHF2 (42%) > PTCH1 (27%) > XPA (20%). LOH was concordant with hemizygous deletion in exonic markers. Homozygous deletions were restricted to PHF2 exon-18 and FANCC exon-2. Interstitial deletion or loss of entire region was observed in 32% (15/47) group-A and 42% (25/59) group-B samples indicating the importance of this region in BC.

Deletion between PTCH1 and XPA was significantly associated in both group-A (P = 0.02) and group-B (P = 0.00003) [Table 3].

**Table 3:** Association between deletions of the 4 candidate genes in early- and late-onset BC

		**Group-A**								**Group-B**							
			
		**PHF2**		**FANCC**		**PTCH1**		**XPA**		**PHF2**		**FANCC**		**PTCH1**		**XPA**	
			
		**D+**	**D-**	**D+**	**D-**	**D+**	**D-**	**D+**	**D-**	**D+**	**D-**	**D+**	**D-**	**D+**	**D-**	**D+**	**D-**
	
**PHF2**	**D+**	-	-	9	11	6	14	6	14	-	-	13	12	5	20	6	19
							
	**D-**			13	14	8	19	4	23			16	18	11	23	6	28
	
**p value**		-		0.83		0.98		0.21		-		0.71		0.29		0.55	
	
**FANCC**	**D+**	-	-	-	-	5	17	5	17	-	-	-	-	7	22	5	24
											
	**D-**					9	16	5	20					9	21	7	23
	
**p value**		-		-		0.32		0.82		-		-		0.61		0.56	
	
**PTCH1**	**D+**	-	-	-	-	-	-	6	8	-	-	-	-	-	-	9	7
															
	**D-**							4	29							3	40
	
**p value**		-		-		-		0.02*		-		-		-		0.00003*	

Abbreviations used are: D+: Deletion positive; D-: Deletion negative; * indicates p value significance

### Promoter Methylation analysis

**Figure 3:**
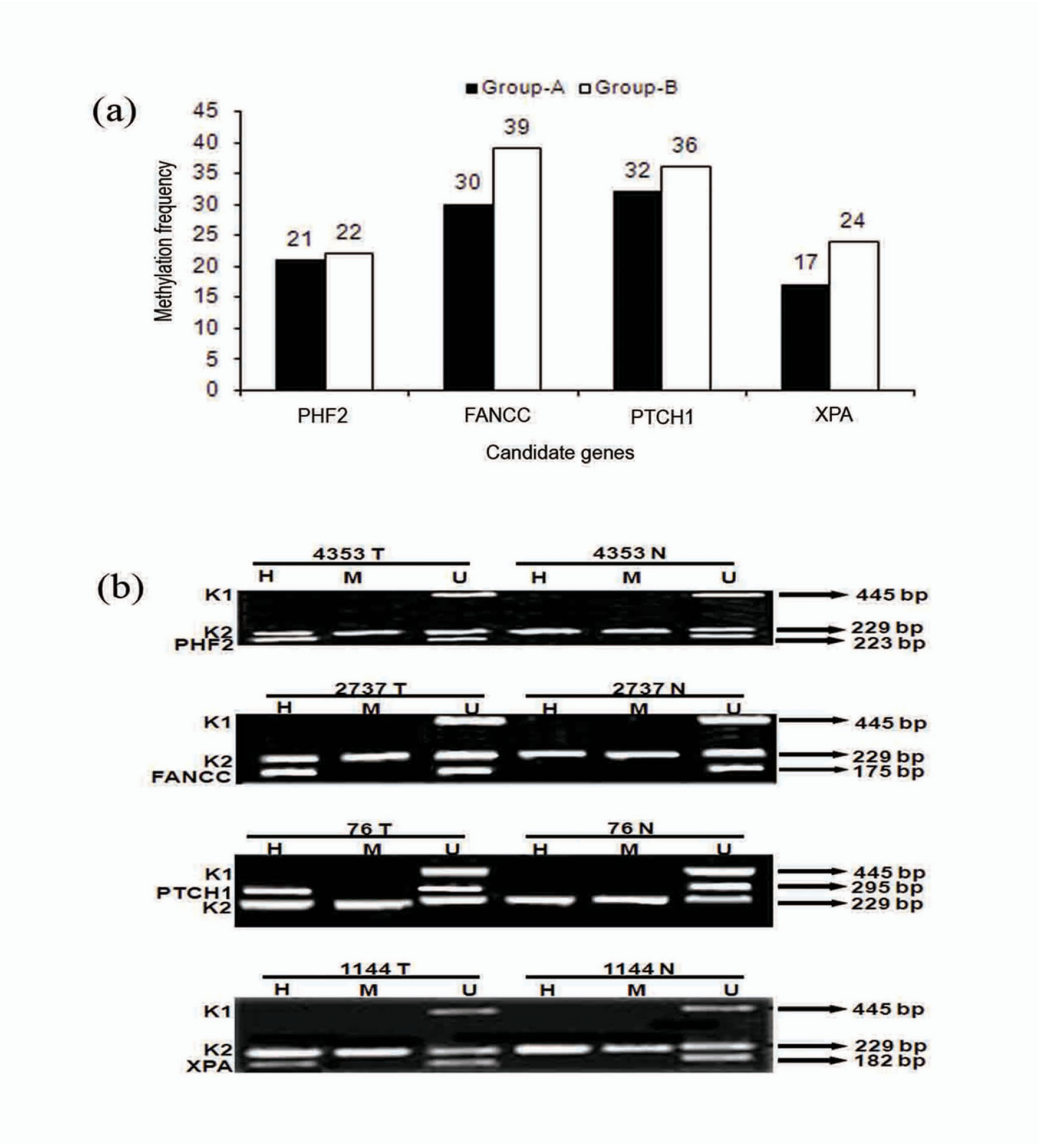
**(a) Histogram showing pattern of methylation in the four genes.** (b) Representative agarose gels showing methylation in PHF2, FANCC, PTCH1 and XPA done by MSRA. H, HpaII digested DNA; M, MspI digested DNA; U, undigested DNA. Controls: A 445-bp fragment of β3A adaptin gene (K1) and 229-bp fragment of RARβ2 exon-1 (K2).

Comparable frequencies of promoter methylation were observed in both age groups. In group-A, PTCH1 exhibited highest 32% methylation followed by FANCC (30%), PHF2 (21%) and XPA (17%). Contrastingly, in group-B, FANCC showed highest 39% methylation followed by 36% in PTCH1, 24% in XPA and 22% in PHF2 [Figure 3; Additional File 3]. Concomitant methylation in all the candidate genes was observed in two early-onset and five late-onset BC samples.

Significant association was observed between methylation of FANCC and PHF2 in both group-A (P = 0.02) and group-B (P = 0.001). Methylation of XPA was significantly associated with methylation of PHF2 (P = 0.03) and FANCC (P = 0.0001) in group-A and that of PTCH1 (P = 0.01) in group-B [Table 4]. In FANCC, both deletion and methylation showed significant association in both groups (P = 0.004 and 0.002) while, in PTCH1 significant association was observed only in group-A (P = 0.002) [data no shown].

**Table 4:** Association between methylation of the 4 candidate genes in early- and late-onset BC

		**Group-A**								**Group-B**							
			
		**PHF2**		**FANCC**		**PTCH1**		**XPA**		**PHF2**		**FANCC**		**PTCH1**		**XPA**	
			
		**M+**	**M-**	**M+**	**M-**	**M+**	**M-**	**M+**	**M-**	**M+**	**M-**	**M+**	**M-**	**M+**	**M-**	**M+**	**M-**
	
**PHF2**	**M+**	-	-	6	4	2	8	4	6	-	-	10	3	7	6	5	8
							
	**M-**			8	29	13	24	4	33			13	33	14	32	9	37
	
**p value**		-		0.02*		0.36		0.03*		-		0.001*		0.12		0.16	
	
**FANCC**	**M+**	-	-	-	-	6	8	7	7	-	-	-	-	7	16	7	16
											
	**M-**					9	24	1	32					14	22	7	29
	
**p value**		-		-		0.29		0.0001*		-		-		0.51		0.33	
	
**PTCH1**	**M+**	-	-	-	-	-	-	4	11	-	-	-	-	-	-	9	12
															
	**M-**							4	28							5	33
	
**p value**		-		-		-		0.23		-		-		-		0.01*	

Abbreviations used are: M+: Methylation positive; M-: Methylation negative; * indicates p value significance

### Mutation analysis of PTCH1 Exon-23

SSCP analysis in 60 primary BC samples revealed no abnormal band shifts in PTCH1 exon-23. Direct sequencing of 20 out of these 60 samples showed no changes in the nucleotide sequences.

### Association between overall alterations of candidate TSGs

**Figure 4:**
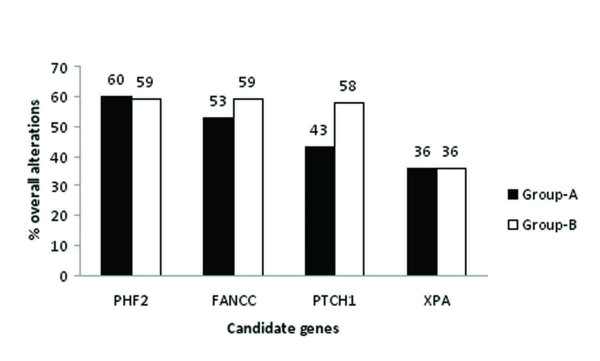
Patten of overall alterations in 4 candidate genes at chr9q22.32-22.33.

Either deletion or methylation of any one of the 4 candidate genes were seen in about 91% of the samples in both groups indicating the importance of chr.9q22.32-22.33 in the development of BC. Overall alterations (deletion/methylation) of PHF2, FANCC, PTCH1 and XPA were comparable in both age groups, them showing 60%, 53%, 43% and 36% alterations respectively in group-A and 59%, 59%, 58% and 36% in group-B (Figure 4; Additional File 4). Overall alterations of PTCH1 showed significant association with XPA in both group-A (P = 0.003) and group-B (P = 0.007). However, overall alterations of PHF2 showed significant association with either FANCC (P = 0.005) or XPA (P = 0.05) only in group-B [data not shown].

### Expression analysis of the candidate genes

**Figure 5:**
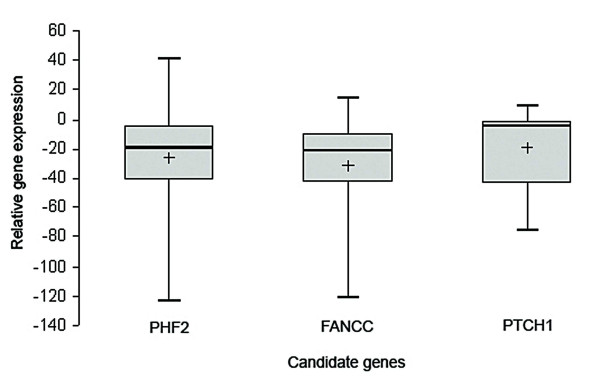
**Box plot representing the relative expression level of PHF2, FANCC and PTCH1 genes done by Q-PCR as shown in the *y*-axis.** Each box shows the distribution of expression levels from 25th to 75th percentile. The median is shown as a line across the box, whereas the '+' is the calculated mean expression level for the particular subtype. Overall, the cases consistently had lower expression of the candidate TSGs.

**Figure 6:**
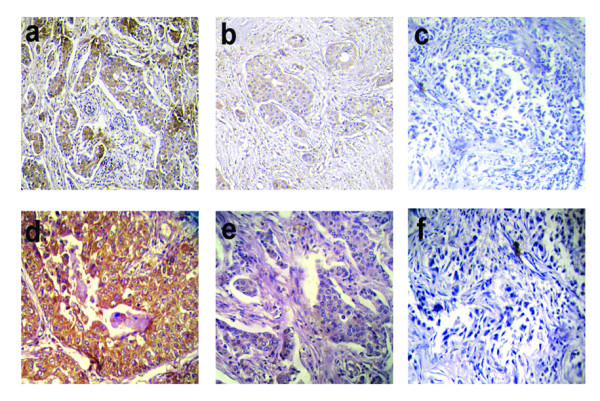
**Immunohistochemistry of FANCC and PTCH1 (a-c) FANCC expression pattern (d-f) PTCH1 expression pattern.** BC samples showing high (a and d) or moderate (b and e) or low (c and f) expression of FANCC and PTCH1 genes respectively. Arrow (→) indicates the expression pattern of FANCC and PTCH1 in primary BC. All magnifications are at 20×.

Q-PCR analysis of PHF2, FANCC and PTCH1 mRNA revealed reduced mRNA expression of in all these genes. In respect to non-tumor control, relative fold reduction in expression of these genes in primary tumors was seen in the following order: FANCC (31.6 ± 36.7) > PHF2 (26.54 ± 44.41) > PTCH1 (19.3 ± 27.8). Reduced expression was noted in nearly 33–40% of the tumors, greater than the mean fold in reduction of the respective genes [Figure 5; Additional File 5]. Immunohistochemical analysis showed both FANCC [Figure 6a–c] and PTCH1 proteins [Figure 6d–f] to be localized exclusively within the cytoplasm. Strong cytoplasmic staining in genes observed in samples with no alterations (deletion and/or methylation) of the candidate genes, while moderate to negative expression was observed in samples harboring genetic and/or epigenetic alterations. Table 5 indicates significant association between alterations of FANCC (P = 0.004) and PTCH1 (P = 0.001) genes with their protein expression as seen by immunohistochemistry. The immunohistochemical analysis of PHF2 was not done due to the lack of commercially available antibody.

**Table 5:** Immunohistochemical expression of FANCC and PTCH1 with respect to their alterations

	**FANCC alteration**		**PTCH1 alteration**	
				
**Sample No.**	**Deletion/Methylation**	**FANCC protein expression**	**Deletion/Methylation**	**PTCH1 protein expression**
1186	+/-	-	-/-	+++
374	-/+	-	-/+	-
4187	+/-	+	-/+	-
3266	+/+	-	-/-	+++
5451	-/-	+++	-/-	+++
5596	-/-	+++	-/+	-
3156	+/+	-	-/-	+++
4604	+/+	-	+/+	-
4131	-/+	++	+/-	-
2490	+/-	-	-/+	-
2400	+/+	-	-/+	++
796	+/-	++	-/+	-
880	-/-	+++	+/+	-
314	+/-	++	+/-	++
5337	+/-	-	-/-	+++
1865	+/+	-	-/+	-
4671	-/-	+++	+/-	+
3025	+/+	-	-/+	-
6155	-/+	-	-/-	+++
1144	-/-	+++	-/+	-

**P value**	P = 0.004 *		P = 0.001 *	

Abbreviations used are: Sample no is patient's registration number

### Association with some DNA repair genes

Of the 106 BC samples a subset of 29 group-A and 33 group-B breast samples were further studied to find out the association between the candidate genes with some breast cancer susceptibility genes like BRCA1, BRCA2 that are intermittently associated with breast cancer. Deletion status of BRCA1 and BRCA2 in the 29 group-A and 33 group-B samples has already been reported [42]. Overall alterations of FANCC showed significant association with deletion of BRCA2 in both group-A (P = 0.03) and group-B (P = 0.02). However, only in group-A, significant association was observed between overall alterations of PTCH1 and deletion of BRCA1 (P = 0.04) [Additional File 6].

### Clinico-pathological association and patient survival

In group-A, both deletion and methylation of PTCH1 were significantly associated with advanced stages. Moreover, its deletion in group-A and methylation in group-B were significantly associated with higher tumor grades (P = 0.02) and lymph node metastasis (P = 0.03) respectively. Methylation of XPA also showed significant association with lymph node invasion (P = 0.04) in group-A [Additional Files 7 and 8].

**Figure 7:**
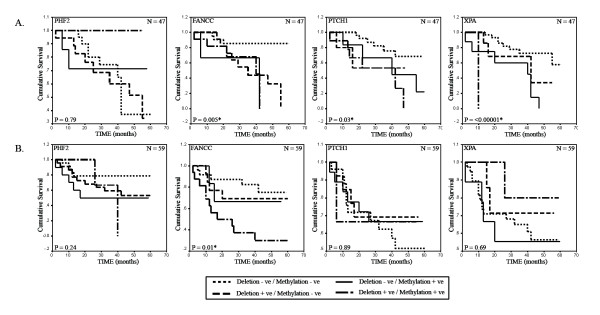
**Kaplan-Meier 5-year survival probability curves with cumulative survival of BC patients by alteration status of PHF2, FANCC, PTCH1 and XPA in (A.) early-onset and (B.) late-onset BC.** Survival time was defined as the time from surgery to the patient's death, known recurrence or the last time the patient was known to be alive. The log rank test was used to assess the differences in the patient survival between cases with plot for the differences in overall survival among cases with any alteration (Deletion +ve/Alteration -ve or Deletion -ve/Methylation +ve or Deletion +ve/Methylation +ve) and no alteration (Deletion -ve/Methylation -ve) of the analyzed genes. *N * denotes sample size.

The clinical outcome in the two age groups was investigated for a period of 5 years. The patient follow-up ranged from 2–60 months in both age groups. The mean and median follow-up period in group-A was 32 months each while, in group-B it was 35 months and 39 months respectively. Log-rank test uncovered statistically significant differences in overall patient survival between cases with presence and absence of any alteration (deletion and/or methylation) in FANCC (P = 0.005), PTCH1 (P = 0.03) and XPA (P = < 0.00001) among group-A samples. However in group-B samples, alterations of only FANCC (P = 0.01) was significantly associated with survival [Figure 7].

## Discussion

Previous studies have shown differences in alteration pattern of various chromosomal regions in the development of early- and late-onset BC [3-6]. To delineate the molecular pathogenesis of BC in the two age groups in detail, molecular alterations (deletion/methylation) of candidate genes PHF2, FANCC, PTCH1 and XPA within chr.9q22.32-22.33 region were analyzed due to its association with a wide variety of tumors including BC. Nearly 91% samples in both groups presented alterations in any one of the genes. Compared to XPA, overall frequencies of alterations in PHF2, FANCC and PTCH1 were higher (Figure 4). This is the first report of its kind. The overall alterations of these genes were differentially associated in the two age groups and indicated differences in pathogenesis and adverse prognosis in patients.

Significant association between deletion and methylation of FANCC in both groups and PTCH1 in group-A only, suggested them to be candidate TSGs in BC. The concordance between deletion/methylation of these genes with protein expression also supported this fact. Down-regulation in mRNA expression of these genes were also seen in MCF-7 and primary BC samples. Deletion, mutation and down-regulation of FANCC have also been reported in other malignancies [16-22]. Significant association of FANCC-XPA methylation in group-A and FANCC-BRCA2 deletion in both age groups supported interlink between multiple DNA repair pathways. This warrants further analysis of other members of this repair pathway needs to be done. The association noted between FANCC alterations and adverse patient survival in either age group suggests it as a prognostic marker.

Contrary to previous reports, frequencies of alterations in PTCH1 in our samples were higher [9]. This may be due to differences in etiology, ethnicity and techniques used in our study. Mutations in exon-23 of PTCH1 were reported in BCC but, no such changes were seen in our samples [43]. Significant association between PTCH1 and XPA deletion in both groups as well as between their methylation in group-B suggested some functional relationship between hedgehog dependent stem cell renewal pathway and the NER pathway. Coincident germline mutations in PTCH1 and BRCA1 genes have been seen in familial BC and NBCCS [24]. Similarly, correlation between PTCH1 alterations with BRCA1 deletion in group-A suggested its association with other DNA repair pathways. Poor survival in group-A patients with PTCH1 alterations indicated its prognostic significance. In PHF2 and XPA no association was seen between their deletion and methylation. So, other mechanisms of inactivation such as mutation cannot be ruled out. High deletion along with significant association of PHF2 methylation with FANCC methylation in both age groups and XPA methylation in group-A suggested PHF2 to be a candidate TSGs in BC. Reduced expression of PHF2 mRNA also supported this fact. Further analysis of PHF2 is required to assess its role in tumorigenesis.

## Conclusion

Thus it may be concluded that impairment of multiple DNA repair pathways and PTCH1 associated self-renewal pathway are necessary for the development of both groups of BC. However, differential association of these genes with adverse patient outcome in both groups suggests their differences in molecular pathogenesis.

## Abbreviations

ATCC: American Type Culture Collection; BC: Breast carcinoma; BCC: basal cell carcinoma; CGH: Comparative Genomic Hybridization; Chr.: Chromosome; HD: Homozygous deletion; HED: Hemizygous deletion; IHC: Immunohistochemisty; LOH: loss of heterozygosity; MSRA: Methylation Sensitive Restriction Analysis; NBCCS: nevoid basal cell carcinoma syndrome; NER: nucleotide excision repair; Q-PCR: Quantitative Real-time Polymerase Chain Reaction; SCC: squamous cell carcinoma; UICC: International Union against Cancer

## Competing interests

The authors declare that they have no competing interests.

## Authors' contributions

SS carried out the molecular genetic studies, participated in the sequence alignment and drafted the manuscript. RKS participated in the design of the study and performed the statistical analysis. NA provided the samples needed for this study. AR carried the histological studies. SR participated in its design and coordination and helped to draft the manuscript. CKP conceived of the study, and participated in its design and coordination and helped to draft the manuscript.

## Supplementary Material

Additional file 1 **Pattern of alterations in early-onset (≤ 40 years) breast carcinoma.** Click here for file

Additional file 2 **Pattern of alterations in late-onset (> 40 years) breast carcinoma.** Click here for file

Additional file 3 **Frequency of methylation of chr.9q22.32-22.33 candidate genes in early- and late-onset breast carcinoma.** Click here for file

Additional file 4 **Frequency of overall alterations (deletion/methylation) of candidate TSGs in breast carcinoma.** Click here for file

Additional file 5 **Q-PCR data sheet for expression analysis of candidate TSGs.** Click here for file

Additional file 6 **Association between overall alterations of the candidate TSGs at chr.9q22.32-22.33 and other DNA repair genes in early- and late-onset BC.** Click here for file

Additional file 7 **Clinico-pathological correlation of deletion in different genes at chr.9q22.32-22.33 in Group-A and Group-B breast carcinomas.** Click here for file

Additional file 8 **Clinico-pathological correlation of methylation in different genes at chr.9q22.32-22.33 in Group-A and Group-B breast carcinomas.** Click here for file
