# Supplementary material for: Alterations in candidate genes PHF2, FANCC, PTCH1 and XPA at chromosomal 9q22.3 region: Pathological significance in early- and late-onset breast carcinoma
Source: Mol Cancer. 2008 Nov 6;7:84. doi: 10.1186/1476-4598-7-84 (PMC2633285; doi:10.1186/1476-4598-7-84)
Supplement: Additional file 6 — Association between overall alterations of the candidate TSGs at chr.9q22.32-22.33 and other DNA repair genes in early- and late-onset BC. [file 1476-4598-7-84-S6.doc]

|  |  | **EARLY-ONSET BC (N = 29)** | | | | | | | | | | | |  | **LATE-ONSET BC (N = 33)** | | | | | | | | | | | |
| --- | --- | --- | --- | --- | --- | --- | --- | --- | --- | --- | --- | --- | --- | --- | --- | --- | --- | --- | --- | --- | --- | --- | --- | --- | --- | --- |
|  | | **PHF2** | | **FANCC** | | **PTCH** | | **XPA** | | **BRCA1** | | **BRCA2** | |  | **PHF2** | | **FANCC** | | **PTCH** | | **XPA** | | **BRCA1** | | **BRCA2** | |
|  |  | **A+** | **A-** | **A+** | **A-** | **A+** | **A-** | **A+** | **A-** | **A+** | **A-** | **A+** | **A-** |  | **A+** | **A-** | **A+** | **A-** | **A+** | **A-** | **A+** | **A-** | **A+** | **A-** | **A+** | **A-** |
| **PHF2** | **A+** | - | - | 11 | 6 | 6 | 11 | 6 | 11 | 12 | 5 | 11 | 6 |  | - | - | 15 | 6 | 14 | 7 | 11 | 10 | 11 | 10 | 11 | 10 |
|  | **A-** |  |  | 7 | 5 | 5 | 7 | 3 | 9 | 10 | 2 | 7 | 5 |  |  |  | 3 | 9 | 6 | 6 | 2 | 10 | 7 | 5 | 3 | 9 |
| P value | | na | | 0.73 | | 0.73 | | 0.56 | | 0.43 | | 0.73 | |  | na | | **0.01*** | | 0.36 | | **0.04*** | | 0.74 | | 0.13 | |
| **FANCC** | **A+** | - | - | - | - | 7 | 11 | 8 | 10 | 15 | 3 | 14 | 4 |  | - | - | - | - | 12 | 6 | 9 | 9 | 12 | 6 | 11 | 7 |
|  | **A-** |  |  |  |  | 4 | 7 | 1 | 10 | 7 | 4 | 4 | 7 |  |  |  |  |  | 8 | 7 | 4 | 11 | 6 | 9 | 3 | 12 |
| P value | | na | | na | | 0.89 | | **0.05*** | | 0.23 | | **0.03*** | |  | na | | na | | 0.44 | | 0.17 | | 0.13 | | **0.02*** | |
| **PTCH** | **A+** | - | - | - | - | - | - | 6 | 5 | 6 | 5 | 6 | 5 |  | - | - | - | - | - | - | 13 | 7 | 12 | 8 | 8 | 12 |
|  | **A-** |  |  |  |  |  |  | 3 | 15 | 16 | 2 | 12 | 6 |  |  |  |  |  |  |  | 0 | 13 | 6 | 7 | 6 | 7 |
| P value | | na | | na | | na | | **0.03*** | | **0.04*** | | 0.51 | |  | na | | na | | na | | **0.0001*** | | 0.44 | | 0.73 | |
| **XPA** | **A+** | - | - | - | - | - | - | - | - | 5 | 4 | 6 | 3 |  | - | - | - | - | - | - | - | - | 7 | 6 | 6 | 7 |
|  | **A-** |  |  |  |  |  |  |  |  | 17 | 3 | 12 | 8 |  |  |  |  |  |  |  |  |  | 11 | 9 | 8 | 12 |
| P value | | na | | na | | na | | na | | 0.09 | | 0.73 | |  | na | | na | | na | | na | | 0.95 | | 0.73 | |
| **BRCA1** | **A+** | - | - | - | - | - | - | - | - | - | - | 14 | 8 |  | - | - | - | - | - | - | - | - | - | - | 10 | 8 |
|  | **A-** |  |  |  |  |  |  |  |  |  |  | 4 | 3 |  |  |  |  |  |  |  |  |  |  |  | 14 | 1 |
| P value | | na | | na | | na | | na | | na | | 0.76 | |  | na | | na | | na | | na | | na | | **0.01*** | |

**Additional File 6:** Association between overall alterations of the candidate TSGs at chr.9q22.32-22.33 and other DNA repair genes in early- and late-onset BC

Abbreviations used are: **A+**: Alteration positive; **A-**: Alteration negative; * indicates p value significance.
