# Supplementary material for: Alterations in candidate genes PHF2, FANCC, PTCH1 and XPA at chromosomal 9q22.3 region: Pathological significance in early- and late-onset breast carcinoma
Source: Mol Cancer. 2008 Nov 6;7:84. doi: 10.1186/1476-4598-7-84 (PMC2633285; doi:10.1186/1476-4598-7-84)
Supplement: Additional file 7 — Clinico-pathological correlation of deletion in different genes at chr.9q22.32-22.33 in Group-A and Group-B breast carcinomas. [file 1476-4598-7-84-S7.doc]

**Additional File 7:** Clinico-pathological correlation of deletion in different genes at chr.9q22.32-22.33 in Group-A

and Group-B breast carcinomas

| Group A | **Deletion** | | | | | | | | | | | | | | | |
| --- | --- | --- | --- | --- | --- | --- | --- | --- | --- | --- | --- | --- | --- | --- | --- | --- |
| **Clinical parameter** | **Locus** | | | | | | | | | | | | | | | |
|  | **PHF2** | | **Total** | **Overall** | **FANCC** | | **Total** | **Overall** | **PTCH1** | | **Total** | **Overall** | **XPA** | | **Total** | **Overall** |
|  | **D+** | **D-** |  | **p value** | **D+** | **D-** |  | **p value** | **D+** | **D-** |  | **p value** | **D+** | **D-** |  | **p value** |
| **Grade I** | 3 | 1 | 4 | 0.17 | 1 | 3 | 4 | 0.95 | 4 | 0 | 4 | **0.02*** | 1 | 3 | 4 | 0.75 |
| **Grade II** | 17 | 15 | 32 |  | 16 | 16 | 32 |  | 8 | 24 | 32 |  | 7 | 25 | 32 |  |
| **Grade III** | 4 | 7 | 11 |  | 5 | 6 | 11 |  | 2 | 9 | 11 |  | 2 | 9 | 11 |  |
| **Stage I+II** | 4 | 4 | 8 | 0.64 | 2 | 6 | 8 | 0.17 | 5 | 3 | 8 | **0.03*** | 2 | 6 | 8 | 0.78 |
| **Stage III+IV** | 16 | 23 | 39 |  | 20 | 19 | 39 |  | 9 | 30 | 39 |  | 8 | 31 | 39 |  |
| **Lymph node -** | 6 | 8 | 14 | 0.82 | 6 | 8 | 14 | 0.26 | 4 | 10 | 14 | 0.91 | 3 | 11 | 14 | 0.99 |
| **Lymph node +** | 13 | 20 | 33 |  | 16 | 17 | 33 |  | 10 | 23 | 33 |  | 7 | 26 | 33 |  |
|  |  |  |  |  |  |  |  |  |  |  |  |  |  |  |  |  |
|  |  |  |  |  |  |  |  |  |  |  |  |  |  |  |  |  |
| Group B | **Deletion** | | | | | | | | | | | | | | | |
| **Clinical parameter** | **Locus** | | | | | | | | | | | | | | | |
|  | **PHF2** | | **Total** | **Overall** | **FANCC** | | **Total** | **Overall** | **PTCH1** | | **Total** | **Overall** | **XPA** | | **Total** | **Overall** |
|  | **D+** | **D-** |  | **p value** | **D+** | **D-** |  | **p value** | **D+** | **D-** |  | **p value** | **D+** | **D-** |  | **p value** |
| **Grade I** | 4 | 4 | 8 | 0.84 | 2 | 6 | 8 | 0.35 | 3 | 5 | 8 | 0.29 | 2 | 6 | 8 | 0.92 |
| **Grade II** | 13 | 26 | 39 |  | 20 | 19 | 39 |  | 11 | 28 | 39 |  | 7 | 32 | 39 |  |
| **Grade III** | 6 | 6 | 12 |  | 6 | 6 | 12 |  | 2 | 10 | 12 |  | 3 | 9 | 12 |  |
| **Stage I+II** | 12 | 11 | 23 | 0.22 | 11 | 12 | 23 | 0.87 | 6 | 17 | 23 | 0.89 | 4 | 19 | 23 | 0.65 |
| **Stage III+IV** | 13 | 23 | 36 |  | 18 | 18 | 36 |  | 10 | 26 | 36 |  | 8 | 28 | 36 |  |
| **Lymph node -** | 4 | 7 | 11 | 0.65 | 6 | 5 | 11 | 0.69 | 3 | 8 | 11 | 0.99 | 3 | 8 | 11 | 0.53 |
| **Lymph node +** | 21 | 27 | 48 |  | 23 | 25 | 48 |  | 13 | 35 | 48 |  | 9 | 39 | 48 |  |

Abbreviations used are: **D+**: Deletion positive; **D-**: Deletion negative; * indicates p value significance.
