# Supplementary material for: Alterations in candidate genes PHF2, FANCC, PTCH1 and XPA at chromosomal 9q22.3 region: Pathological significance in early- and late-onset breast carcinoma
Source: Mol Cancer. 2008 Nov 6;7:84. doi: 10.1186/1476-4598-7-84 (PMC2633285; doi:10.1186/1476-4598-7-84)
Supplement: Additional file 8 — Clinico-pathological correlation of methylation in different genes at chr.9q22.32-22.33 in Group-A and Group-B breast carcinomas. [file 1476-4598-7-84-S8.doc]

**Additional File 8:** Clinico-pathological correlation of methylation in different genes at chr.9q22.32-22.33 in Group-A

and Group-B breast carcinomas

| Group A | **Methylation** | | | | | | | | | | | | | | | |
| --- | --- | --- | --- | --- | --- | --- | --- | --- | --- | --- | --- | --- | --- | --- | --- | --- |
| **Clinical parameter** | **Locus** | | | | | | | | | | | | | | | |
|  | **PHF2** | | **Total** | **Overall** | **FANCC** | | **Total** | **Overall** | **PTCH1** | | **Total** | **Overall** | **XPA** | | **Total** | **Overall** |
|  | **M+** | **M-** |  | **p value** | **M+** | **M-** |  | **p value** | **M+** | **M-** |  | **p value** | **M+** | **M-** |  | **p value** |
| **Grade I** | 2 | 2 | 4 | 0.11 | 1 | 3 | 4 | 0.27 | 1 | 3 | 4 | 0.48 | 0 | 4 | 4 | 0.89 |
| **Grade II** | 7 | 25 | 32 |  | 8 | 24 | 32 |  | 12 | 20 | 32 |  | 7 | 25 | 32 |  |
| **Grade III** | 1 | 10 | 11 |  | 5 | 6 | 11 |  | 2 | 9 | 11 |  | 1 | 10 | 11 |  |
| **Stage I+II** | 1 | 7 | 8 | 0.51 | 3 | 5 | 8 | 0.6 | 5 | 3 | 8 | **0.04*** | 3 | 5 | 8 | 0.09 |
| **Stage III+IV** | 9 | 30 | 39 |  | 11 | 28 | 39 |  | 10 | 29 | 39 |  | 5 | 34 | 39 |  |
| **Lymph node -** | 1 | 13 | 14 | 0.12 | 1 | 13 | 14 | 0.03 | 5 | 9 | 14 | 0.72 | 0 | 14 | 14 | **0.04*** |
| **Lymph node +** | 9 | 24 | 33 |  | 13 | 20 | 33 |  | 10 | 23 | 33 |  | 8 | 25 | 33 |  |
|  |  |  |  |  |  |  |  |  |  |  |  |  |  |  |  |  |
|  |  |  |  |  |  |  |  |  |  |  |  |  |  |  |  |  |
| Group B | **Methylation** | | | | | | | | | | | | | | | |
| **Clinical parameter** | **Locus** | | | | | | | | | | | | | | | |
|  | **PHF2** | | **Total** | **Overall** | **FANCC** | | **Total** | **Overall** | **PTCH1** | | **Total** | **Overall** | **XPA** | | **Total** | **Overall** |
|  | **M+** | **M-** |  | **p value** | **M+** | **M-** |  | **p value** | **M+** | **M-** |  | **p value** | **M+** | **M-** |  | **p value** |
| **Grade I** | 0 | 8 | 8 | 0.23 | 1 | 7 | 8 | 0.12 | 3 | 5 | 8 | 0.58 | 2 | 6 | 8 | 0.58 |
| **Grade II** | 9 | 30 | 39 |  | 16 | 23 | 39 |  | 11 | 28 | 39 |  | 8 | 31 | 39 |  |
| **Grade III** | 3 | 9 | 12 |  | 6 | 6 | 12 |  | 7 | 5 | 12 |  | 4 | 8 | 12 |  |
| **Stage I+II** | 5 | 18 | 23 | 0.83 | 9 | 14 | 23 | 0.99 | 8 | 15 | 23 | 0.92 | 6 | 17 | 23 | 0.73 |
| **Stage III+IV** | 7 | 29 | 36 |  | 14 | 22 | 36 |  | 13 | 23 | 36 |  | 8 | 28 | 36 |  |
| **Lymph node -** | 1 | 10 | 11 | 0.3 | 3 | 8 | 11 | 0.38 | 7 | 4 | 11 | **0.03*** | 5 | 6 | 11 | 0.06 |
| **Lymph node +** | 11 | 37 | 48 |  | 20 | 28 | 48 |  | 14 | 34 | 48 |  | 9 | 39 | 48 |  |

Abbreviations used are: **M+**: Methylation positive; **M-**: Methylation negative; * indicates p value significance.
